# Supplementary material for: LinAliFold and CentroidLinAliFold: fast RNA consensus secondary structure prediction for aligned sequences using beam search methods
Source: Bioinform Adv. 2022 Oct 22;2(1):vbac078. doi: 10.1093/bioadv/vbac078 (PMC9710674; doi:10.1093/bioadv/vbac078)
Supplement: vbac078_Supplementary_Data [file vbac078_supplementary_data.pdf]

Supplementary Materials for LinAliFold and CentroidLinAliFold: Fast RNA consensus secondary structure prediction for aligned sequences using beam search methods

Tsukasa Fukunaga and Michiaki Hamada

Supplementary Methods

Further differences between the LinAliFold algorithm and previous tools

LinAliFold is an algorithm that applies a beam search method in LinearFold to RNAalifold, but LinAliFold has three other minor differences from RNAalifold and LinearFold.

The first is the calculation method of hairpin candidates. While LinearFold calculates b hairpin candidates, LinAliFold calculates hairpin candidates with a loop length of b bases or less. In other words, LinAliFold adopts a local folding method in the calculation of hairpin loops. This is because it was difficult to efficiently enumerate b hairpin candidates in the RNA consensus secondary structure prediction. Note that this change should not significantly impact the prediction because hairpins with large loops are unlikely to occur.

The second is the energy evaluation of unpaired nucleotides in multi loops. While RNAalifold counts the numbers of nucleotides in individual sequences, LinAliFold approximates the number of nucleotides by the sub-alignment length in multi loops for fast computation. In other words, RNAaliflod does not include gaps on the multi loops in the numbers of unpaired nucleotides, but LinAliFold includes the gaps in the numbers.

The third is the evaluation of the gaps. While RNAalifold regards gaps as nearby bases when evaluating the type of bases, LinAliFold does not evaluate the gaps in such cases for ease of the implementation.

Supplementary Figures

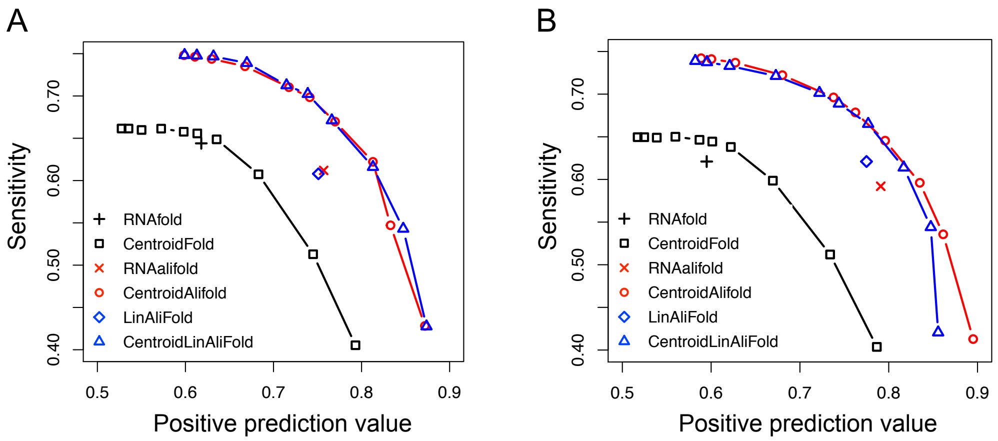

Fig. S1 Prediction performance of RNA secondary structure on the RNAStralign dataset with (A) 5 sequences and (B) 10 sequences. The x-axis and the y-axis represent PPV and SEN, respectively. Symbols and colors used above represent: RNAfold (black crosses), CentroidFold (black squares), RNAalifold (red crosses), CentroidAlifold (red circles), LinAliFold (blue squares), and CentroidLinAliFold (blue triangles).

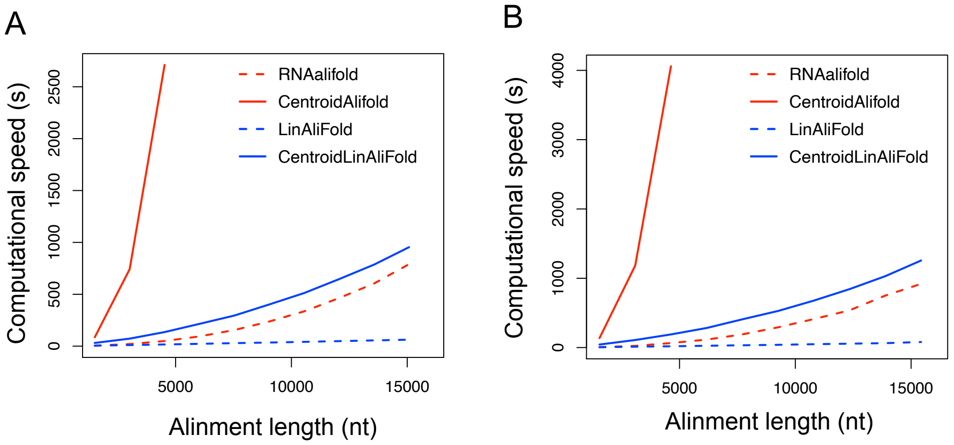

Fig. S2 Evaluation of the computational time for a number of sequences of (A) 5 and (B) 10. The x- and y-axis represent the alignment lengths (nt) and computational time (s), respectively. The lines represent RNAalifold (red dashed), CentroidAlifold (red solid), LinAliFold (blue dashed), and CentroidLinAliFold (blue solid). The exact computational time was not measured when it exceeded 2 hours.

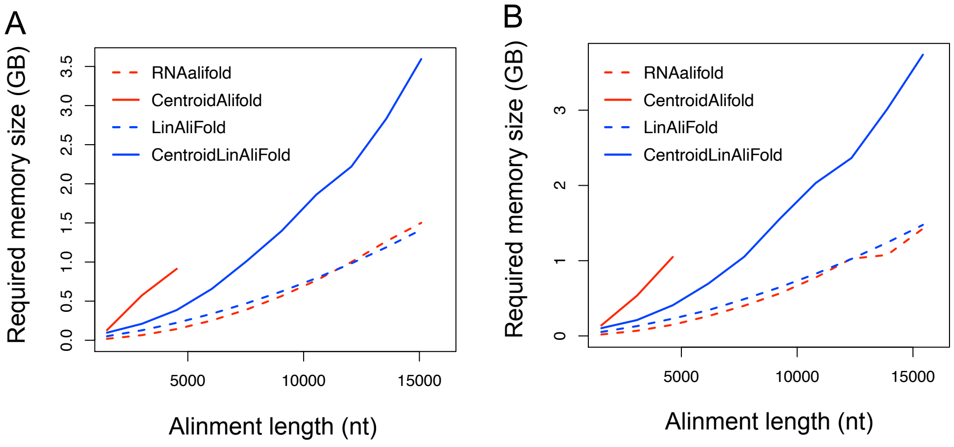

Fig. S3 The required memory size was evaluated for a number of sequences of (A) 5 and (B) 10. The x- and y-axis represent the alignment lengths (nt) and required memory size (GB), respectively. The lines represent RNAalifold (red dashed), CentroidAlifold (red solid), LinAliFold (blue dashed), and CentroidLinAliFold (blue solid). The required memory size was not measured when computational time exceeded 2 hours.

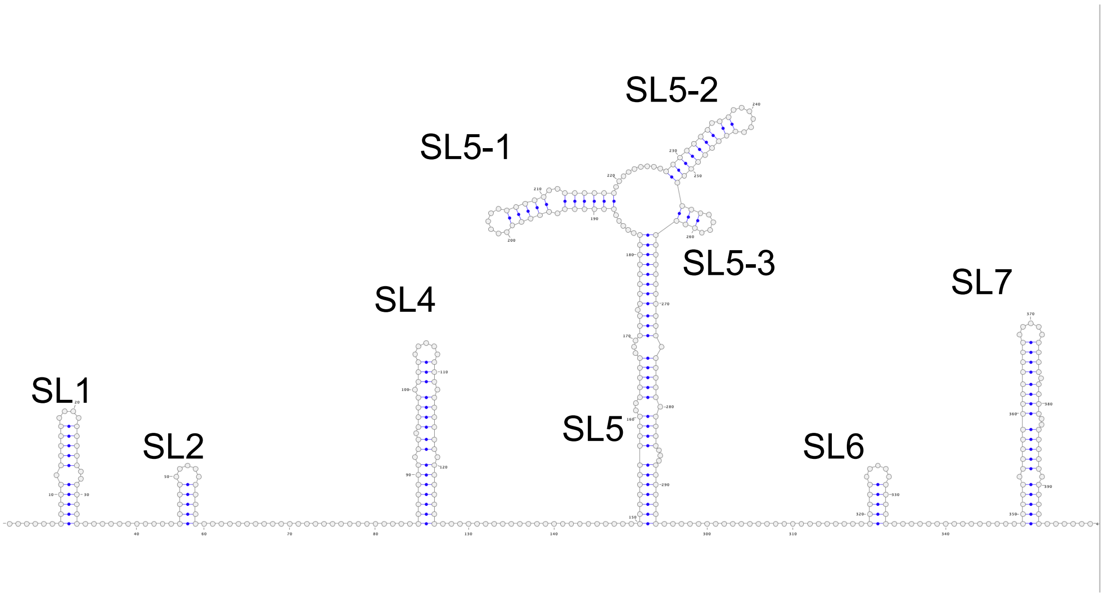

Fig. S4 A predicted RNA consensus secondary structure of coronavirus 5′-UTR by LinAliFold. Graphical view was created using VARNA.

Supplementary Tables

Table S1 The PPV results of prediction accuracy for each RNA family when the number of sequences was 5

RNA Family	RNAfold	CentroidFold	RNAalifold	LinAliFold	CentroidAlifold	CentroidLinAliFold
5s rRNA	0.757	0.742	0.902	0.886	**0.914**	**0.914**
16S rRNA	0.444	0.614	0.686	0.668	**0.877**	0.868
group I intron	0.766	0.843	0.818	0.816	**0.836**	0.835
RNaseP	0.562	0.712	0.685	0.684	**0.782**	0.765
SRP RNA	0.633	0.640	0.684	**0.706**	0.674	0.671
telomerase RNA	0.425	0.503	0.604	0.613	0.705	**0.714**
tmRNA	0.527	0.637	**0.704**	0.659	0.693	**0.704**
tRNA	0.825	0.832	0.973	0.973	0.973	**0.977**
average	0.618	0.691	0.757	0.751	**0.807**	0.806

The bold values are the highest scores in each row.

Table S2 The SEN results of prediction accuracy for each RNA family when the number of sequences was 5

RNA Family	RNAfold	CentroidFold	RNAalifold	LinAliFold	CentroidAlifold	CentroidLinAliFold
5s rRNA	0.760	0.661	**0.890**	0.854	0.868	0.869
16S rRNA	0.448	0.445	0.661	0.643	0.640	**0.668**
group I intron	**0.704**	0.686	0.472	0.488	0.520	0.491
RNaseP	**0.609**	0.556	0.468	0.468	0.482	0.500
SRP RNA	**0.691**	0.652	0.532	0.552	0.559	0.566
telomerase RNA	0.561	0.567	0.572	0.582	0.579	**0.588**
tmRNA	**0.542**	0.529	0.328	0.304	0.389	0.368
tRNA	0.838	0.774	**0.970**	**0.970**	**0.970**	0.937
average	**0.644**	0.609	0.612	0.608	0.626	0.623

The bold values are the highest scores in each row.

Table S3 The MCC results of prediction accuracy for each RNA family when the number of sequences was 5

RNA Family	RNAfold	CentroidFold	RNAalifold	LinAliFold	CentroidAlifold	CentroidLinAliFold
5s rRNA	0.757	0.697	**0.895**	0.869	0.890	0.890
16S rRNA	0.445	0.522	0.673	0.655	0.748	**0.760**
group I intron	0.734	**0.759**	0.615	0.626	0.657	0.637
RNaseP	0.584	**0.627**	0.563	0.562	0.610	0.616
SRP RNA	**0.658**	0.641	0.584	0.606	0.605	0.608
telomerase RNA	0.487	0.533	0.586	0.595	0.635	**0.645**
tmRNA	0.533	**0.577**	0.467	0.436	0.514	0.502
tRNA	0.830	0.799	**0.971**	**0.971**	**0.971**	0.955
average	0.628	0.644	0.669	0.665	**0.704**	0.702

The bold values are the highest scores in each row.

Table S4 The PPV results of prediction accuracy for each RNA family when the number of sequences was 10

RNA Family	RNAfold	CentroidFold	RNAalifold	LinAliFold	CentroidAlifold	CentroidLinAliFold
5s rRNA	0.735	0.732	0.934	0.913	**0.940**	0.919
16S rRNA	0.431	0.584	0.733	0.660	**0.893**	0.836
group I intron	0.732	0.830	0.837	0.853	**0.860**	0.843
RNaseP	0.555	0.727	0.711	0.697	**0.795**	0.787
SRP RNA	0.624	0.647	**0.784**	0.728	0.729	0.726
telomerase RNA	0.407	0.479	0.649	0.659	**0.776**	0.701
tmRNA	0.490	0.599	0.714	**0.723**	0.710	0.706
tRNA	0.785	0.791	**0.964**	**0.964**	**0.964**	**0.964**
average	0.595	0.674	0.791	0.775	**0.833**	0.810

The bold values are the highest scores in each row.

Table S5 The SEN results of prediction accuracy for each RNA family when the number of sequences was 10

RNA Family	RNAfold	CentroidFold	RNAalifold	LinAliFold	CentroidAlifold	CentroidLinAliFold
5s rRNA	0.733	0.660	0.870	**0.889**	0.847	0.867
16S rRNA	0.441	0.440	**0.675**	0.623	0.594	0.595
group I intron	**0.670**	**0.670**	0.481	0.497	0.476	0.498
RNaseP	**0.598**	0.567	0.506	0.529	0.536	0.538
SRP RNA	**0.685**	0.676	0.596	0.625	0.606	0.621
telomerase RNA	0.532	0.537	0.400	**0.552**	0.454	**0.552**
tmRNA	**0.516**	0.501	0.247	0.291	0.332	0.336
tRNA	0.798	0.737	**0.959**	**0.959**	**0.959**	**0.959**
average	**0.622**	0.599	0.592	0.621	0.600	0.621

The bold values are the highest scores in each row.

Table S6 The MCC results of prediction accuracy for each RNA family when the number of sequences was 10

RNA Family	RNAfold	CentroidFold	RNAalifold	LinAliFold	CentroidAlifold	CentroidLinAliFold
5s rRNA	0.732	0.692	**0.900**	**0.900**	0.891	0.892
16S rRNA	0.435	0.506	0.703	0.640	**0.726**	0.704
group I intron	0.700	**0.744**	0.631	0.647	0.637	0.645
RNaseP	0.575	0.640	0.595	0.602	**0.649**	0.646
SRP RNA	0.650	0.657	**0.671**	0.669	0.659	0.665
telomerase RNA	0.464	0.506	0.505	0.599	0.590	**0.617**
tmRNA	0.501	**0.544**	0.416	0.454	0.477	0.478
tRNA	0.789	0.759	**0.961**	**0.961**	**0.961**	**0.961**
average	0.606	0.631	0.673	0.684	0.699	**0.701**

The bold values are the highest scores in each row.

Table S7 The PPV results of prediction accuracy for each RNA family when the number of sequences was 20

RNA Family	RNAfold	CentroidFold	RNAalifold	LinAliFold	CentroidAlifold	CentroidLinAliFold
5s rRNA	0.730	0.716	**0.940**	0.918	**0.940**	0.925
16S rRNA	0.431	0.607	0.726	0.677	**0.892**	0.835
group I intron	0.738	0.836	0.833	0.820	**0.845**	0.823
RNaseP	0.552	0.743	0.748	0.689	0.759	**0.768**
SRP RNA	0.616	0.631	0.770	0.748	**0.775**	0.748
telomerase RNA	0.395	0.461	0.726	0.632	**0.800**	0.715
tmRNA	0.504	0.607	0.744	0.686	**0.749**	0.665
tRNA	0.804	0.815	**0.978**	0.972	**0.978**	0.972
average	0.596	0.677	0.808	0.768	**0.842**	0.806

The bold values are the highest scores in each row.

Table S8 The SEN results of prediction accuracy for each RNA family when the number of sequences was 20

RNA Family	RNAfold	CentroidFold	RNAalifold	LinAliFold	CentroidAlifold	CentroidLinAliFold
5s rRNA	0.732	0.646	0.836	0.879	0.836	**0.884**
16S rRNA	0.438	0.449	**0.645**	0.636	0.607	0.622
group I intron	**0.680**	0.679	0.433	0.492	0.467	0.508
RNaseP	**0.588**	0.568	0.513	0.522	0.536	0.560
SRP RNA	**0.680**	0.659	0.489	0.586	0.495	0.595
telomerase RNA	0.528	**0.532**	0.263	0.491	0.385	0.465
tmRNA	**0.524**	0.500	0.209	0.340	0.291	0.390
tRNA	0.819	0.763	0.948	**0.961**	0.948	**0.961**
average	**0.624**	0.600	0.542	0.614	0.571	0.623

The bold values are the highest scores in each row.

Table S9 The dependencies of the computational speeds (s) on the number of sequences

software	concatenation size	number of sequences 5	10	20
LinAliFold	1	4.77	5.67	7.57
	5	29.19	33.06	44.39
	10	62.60	80.04	143.58
CentroidLinAliFold	1	31.03	43.73	79.09
	5	295.29	407.71	653.50
	10	954.09	1256.32	1892.06

Table S10 The dependencies of the required memory size (GB) on the number of sequences

software	concatenation size	number of sequences 5	10	20
LinAliFold	1	0.05	0.05	0.05
	5	0.47	0.49	0.51
	10	1.41	1.48	1.17
CentroidLinAliFold	1	0.10	0.10	0.11
	5	1.01	1.05	1.11
	10	3.60	3.74	3.79

Table S11 The dependencies of the MCC results on the covariance model when the number of sequences was 20

	RNAalifold		LinAliFold		CentroidLinAlifold	
RNA Family	simple	RIBOSUM	simple	RIBOSUM	simple	RIBOSUM
5s rRNA	0.885	0.919	0.898	0.918	0.903	0.932
16S rRNA	0.684	0.675	0.656	0.658	0.720	0.735
group I intron	0.598	0.621	0.632	0.626	0.644	0.642
RNaseP	0.616	0.685	0.596	0.677	0.654	0.705
SRP RNA	0.596	0.631	0.645	0.634	0.649	0.645
telomerase RNA	0.436	0.573	0.555	0.567	0.572	0.600
tmRNA	0.382	0.510	0.475	0.522	0.505	0.525
tRNA	0.962	0.966	0.966	0.966	0.966	0.966
average	0.645	0.698	0.678	0.696	0.702	0.719

Table S12 The dependencies of the MCC results of LinAliFold on the beam size when the number of sequences was 20

RNA Family	b=10	b=30	b=50	b=100	b=200	b=500
5s rRNA	0.881	0.898	0.898	0.898	0.898	0.898
16S rRNA	0.457	0.613	0.651	0.656	0.714	0.738
group I intron	0.362	0.580	0.609	0.632	0.632	0.632
RNaseP	0.514	0.596	0.596	0.596	0.596	0.596
SRP RNA	0.645	0.645	0.645	0.645	0.645	0.645
telomerase RNA	0.285	0.459	0.490	0.555	0.563	0.563
tmRNA	0.403	0.467	0.475	0.475	0.475	0.475
tRNA	0.936	0.966	0.966	0.966	0.966	0.966
average	0.560	0.653	0.666	0.678	0.686	0.689

Table S13 The dependencies of the MCC results of CentroidLinAliFold on the beam size when the number of sequences was 20

RNA Family	b=10	b=30	b=50	b=100	b=200	b=500
5s rRNA	0.900	0.903	0.903	0.903	0.903	0.903
16S rRNA	0.571	0.666	0.722	0.720	0.771	0.768
group I intron	0.518	0.622	0.640	0.644	0.644	0.644
RNaseP	0.583	0.652	0.654	0.654	0.653	0.653
SRP RNA	0.644	0.649	0.649	0.649	0.649	0.649
telomerase RNA	0.470	0.538	0.557	0.572	0.574	0.575
tmRNA	0.417	0.511	0.504	0.505	0.511	0.511
tRNA	0.944	0.966	0.966	0.966	0.966	0.966
average	0.631	0.688	0.699	0.702	0.709	0.709

Table S14 The dependencies of the computational speeds (s) on the beam size when the number of sequences was 20

		beam size					
software	concatenation size	10	30	50	100	200	500
LinAliFold	1	1.11	2.70	4.24	7.58	12.66	20.13
	5	5.75	15.03	22.95	58.77	91.19	218.62
	10	15.73	38.96	63.16	143.58	261.05	710.48
CentroidLinAliFold	1	11.396	26.21	44.61	79.09	153.56	304.20
	5	156.24	247.67	360.23	653.50	1569.04	5728.0
	10	709.70	941.82	1171.00	1892.06	4245.25	-

'-' represents that the computational time was longer than 2 hours.

Table S15 The dependencies of the required memory size (GB) on the beam size when the number of sequences was 20

software	concatenation size	beam size 10	30	50	100	200	500
LinAliFold	1	0.02	0.03	0.04	0.05	0.08	0.14
	5	0.29	0.34	0.40	0.51	0.69	0.87
	10	1.07	1.17	1.29	1.17	1.76	2.10
CentroidLinAliFold	1	0.07	0.08	0.08	0.11	0.14	0.16
	5	1.07	1.07	1.09	1.11	1.16	2.03
	10	3.43	3.37	3.70	3.79	3.82	-

'-' represents that the computational time was longer than 2 hours.
